# An elastic proteinaceous envelope encapsulates the early *Arabidopsis* embryo

**DOI:** 10.1242/dev.201943

**Published:** 2023-11-09

**Authors:** Yosapol Harnvanichvech, Cecilia Borassi, Diaa Eldin S. Daghma, Hanne M. van der Kooij, Joris Sprakel, Dolf Weijers

**Affiliations:** ^1^ Laboratory of Biochemistry, Wageningen University and Research, Wageningen 6708 WE, The Netherlands; ^2^ Physical Chemistry and Soft Matter, Wageningen University and Research, Wageningen 6708 WE, The Netherlands

**Keywords:** Seed development, Embryo development, Diffusion barrier, Embryonic envelope, *Arabidopsis thaliana*

## Abstract

Plant external surfaces are often covered by barriers that control the exchange of molecules, protect from pathogens and offer mechanical integrity. A key question is when and how such surface barriers are generated. Post-embryonic surfaces have well-studied barriers, including the cuticle, and it has been previously shown that the late *Arabidopsis thaliana* embryo is protected by an endosperm-derived sheath deposited onto a primordial cuticle. Here, we show that both cuticle and sheath are preceded by another structure during the earliest stages of embryogenesis. This structure, which we named the embryonic envelope, is tightly wrapped around the embryonic surface but can be physically detached by cell wall digestion. We show that this structure is composed primarily of extensin and arabinogalactan *O*-glycoproteins and lipids, which appear to form a dense and elastic crosslinked embryonic envelope. The envelope forms in cuticle-deficient mutants and in a mutant that lacks endosperm. This embryo-derived envelope is therefore distinct from previously described cuticle and sheath structures. We propose that it acts as an expandable diffusion barrier, as well as a means to mechanically confine the embryo to maintain its tensegrity during early embryogenesis.

## Teaser

**Summary:** The early *Arabidopsis* embryo is surrounded by a proteinaceous envelope that is distinct from the cuticle and embryo sheath.

## INTRODUCTION

Cells in land plants are generally exposed to the atmosphere, and are often covered by structures that prevent evaporation, exchange of solutes and invasion of pathogens (Nawrath et al., 2013; Yeats and Rose, 2013). These protective structures include the cuticle (Nawrath, 2006; Nawrath et al., 2013), as well as suberin lamellae (Nawrath et al., 2013). In seed plants, the seedling develops from an embryo that is enclosed by maternal tissues during its development (Ingouff et al., 2006; Voiniciuc et al., 2015; Sechet et al., 2018), and the cuticle needs to be complete before seed germination (Yeats and Rose, 2013; De Giorgi et al., 2015; Coen et al., 2019; Creff et al., 2019). In flowering plants, the embryo shares the seed cavity with the product of a second independent fertilization event, the endosperm (Berger, 2003; Hamamura et al., 2012; Ali et al., 2023a,b). As such, the flowering plant embryo surface is exposed to changes occurring in the developing endosperm well before germination, and it is not yet clear to what extent the exchange of materials at the epidermal interface is controlled by a barrier during embryo development. Also, the absence of direct mechanical constraints imposed by the seed coat might mean there is a need for another structure to confine the early embryo during its development. In recent years, a structure that covers the *Arabidopsis thaliana* embryo surface has been described (Fourquin et al., 2016; Moussu et al., 2017; Doll and Ingram, 2022). After double fertilization, the development of the endosperm and the embryo starts, and optimal seed development and germination depend on the communication at the interface between these tissues. At later stages of seed development, the endosperm cellularizes, while the embryo grows invasively into it. At this point, a selective apoplastic lipid-rich barrier, the embryo cuticle, is secreted by the embryonic epidermal cells and limits the communication between the embryo and the endosperm. At the late globular stage, the secretion of cuticle components in vesicles (cutinosomes) starts; these arrange in patches that begin to assemble on the embryo proper surface, and by the heart stage, the cuticle is a continuous layer (Creff et al., 2019). Some molecular components have been identified as key players for the proper assembly of the cutinosomes in a mechanism that requires signaling between the endosperm and the embryo. This pathway involves the endosperm-specific subtilisin protease Abnormal Leaf-shape 1 (ALE1; Xing et al., 2013) and the embryo-expressed receptor kinases GASSHO1 (GSO1) and GASSHO2 (GSO2) (Creff et al., 2019). Later, the embryo continues to grow, and the endosperm is progressively degraded. From the heart stage onwards, endosperm-derived materials accumulate to form a structure on the outer face of the embryo cuticle that is rich in extensin proteins, known as the embryo sheath. This structure is required for normal embryo development, as it prevents adhesion of the embryo to the endosperm during invasive growth. It has been shown that the sheath enables the cotyledons to break through the seed coat, and it acts as the first barrier between the aerial organs and the environment (Doll et al., 2020).

An open question is whether any structure precedes the formation of the embryonic cuticle and embryonic sheath. It could be that no such structure is needed at the early stages, but it might well be that also at the earliest stages, an extracellular structure controls exchange between embryo and endosperm. Also, given that turgor pressure in the endosperm is dynamic throughout seed development (Creff et al., 2023), the embryo might need an extracellular structure to keep its tensegrity.

Here, we report the existence of a previously undescribed extracellular envelope that exists on the surface of the *Arabidopsis* embryo well before the formation of the cuticle and the sheath. The envelope is a protein- and lipid-rich embryo-derived structure, and its biogenesis appears to depend on factors that determine epidermal cell fate. We speculate that the formation of the embryonic envelope could serve as a diffusion barrier that both chemically and physically separates the early embryo from the developing endosperm prior to the assembly of the embryonic cuticle.

## RESULTS

### Early *Arabidopsis* embryos are surrounded by an extracellular structure

**Fig. 1.**
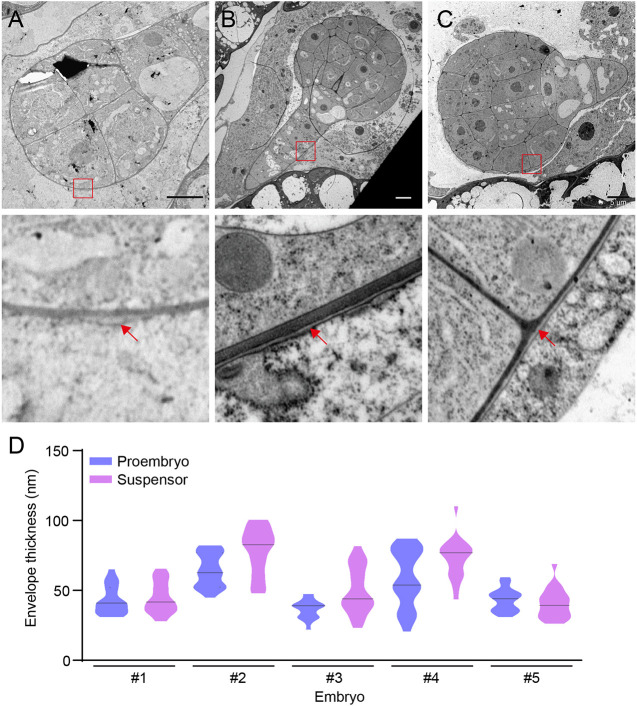
**Ultrastructural analysis of the embryonic envelope.** (A-C) TEM of wild-type *Arabidopsis* embryos at eight-cell and globular stages, showing an electron-dense (white, red arrows) layer around the proembryo and the suspensor. Magnifications of the indicated region are shown in the lower panels. All images are representative of *n*=5 embryos. Scale bars: 5 µm. (D) Quantification of the thickness of the electron-dense (grey or light grey) layer surrounding the pro-embryo and endosperm, measured in sections along the proembryo and suspensor (*n*=5 embryos). A violin plot with the median indicated is shown.

We hypothesized that a structure with barrier-like properties should surround the *Arabidopsis* embryo at early developmental stages. This structure would envelope the embryo, acting as a separation layer between the embryo and the developing endosperm, and it should be flexible enough to accommodate rapid growth. We first performed transmission electron microscopy (TEM) on wild-type seeds. To enable high-definition analysis of native structures, we optimized a method based on encapsulating individual seeds in a mixture of yeast and cyanobacteria (filler), followed by high-pressure freezing and freeze substitution (D.E.S.D, Michael Melzer, Saskia Lippens, Tom Beeckman, Richard S. Smith, Jan van Lent and D.W., unpublished). This method allows ultra-rapid immobilization of cellular processes, thus enabling the study of the three-dimensional organization of subcellular structures. The excellent structural preservation and high resolution allowed a detailed analysis of the embryo surface. Importantly, no rupture of the endosperm–embryo interface occurred during processing. At both the eight-cell and globular stages, we detected a continuous electron-dense layer surrounding both the proembryo and suspensor (Fig. 1A-C). The layer appeared homogeneous along the embryo surface. We measured the cross-sectional thickness in sections from five different embryos ranging from the eight-cell stage to the mid-globular stage. The thickness around the proembryo and along the suspensor ranged from between 40 and 70 nm (Fig. 1D). Hence, the structure is thicker than the plasma membrane but thinner than a primary cell wall.

### Enzymatic liberation of the extra-embryonic envelope

From its appearance in TEM, it is difficult to tell what the envelope structure is made of. We first explored the possibility that this is a cell wall-like structure and treated isolated embryos with a range of cell wall-degrading enzymes. Strikingly, none of the individually tested enzymes or mixtures (cellulase, pectinase, hemicellulase, Driselase and macerozyme), or combinations thereof, led to visual dissociation of embryo cells (Fig. S1A), which would be an indication that any surface layer had degraded. When combining all these enzymes, however, we observed that a translucent layer parted from the embryo surface, creating a ‘halo’ surrounding the embryo (Fig. S1A). We used the same enzyme mixture to test whether this structure was also present in other plant species. We did not find the enzyme treatment led to the liberation of a similar structure in tomato (*Solanum lycopersicum*) or *Brassica napus* zygotic embryos, or from *Brassica* microspore embryo cultures (Fig. S2). The treatment seems to be ineffective in these embryos, although we do not know whether this combination of enzymes is capable of digesting the appropriate linkages in these other species.

**Fig. 2.**
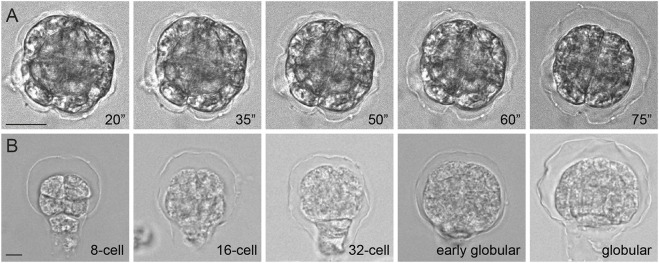
**The embryonic envelope is present from early developmental stages.** (A) Time course of the embryo envelope liberation (representative of *n*=5 embryos). The ″ represents minutes from application of digestion mix. Scale bar: 20 μm. (B) Different stages of *A. thaliana* embryos showing the envelope detached from its surface (representative of *n*≥15 embryos per stage). Scale bar: 10 μm.

Following the same embryo over time showed that the structure was liberated after ∼20 min of incubation (Fig. 2A). Because the embryo shrinks as a consequence of prolonged enzyme treatment (Fig. S1B), the structure becomes more distinctly visible with incubation time, but does not visibly dissociate (Fig. 2A). As seen for the features observed in TEM, this structure is found surrounding both the proembryo and the suspensor. When treating embryos isolated at different stages of development, we found the structure to exist from the earliest time point analyzed (eight-cell stage) onward (Fig. 2B). Although we cannot formally demonstrate that the structure observed in TEM and upon enzyme treatment are the same, we interpret this as visualizations of the same extra-embryonic structure, which we term the ‘embryonic envelope’ (henceforth ‘envelope’). Given that not even an aggressive mixture of enzymes that breaks a range of cell wall linkages can destroy the envelope, we conclude that its main structural component is not of cell wall polysaccharidic nature. The envelope does seem to be linked to the embryo cell walls, as enzymes that degrade the cell wall liberate it. As only a complex mixture of enzymes is effective, the association of the envelope to the embryo cell walls is likely based on interactions or bonds with multiple cell wall polysaccharides.

### The envelope is distinct from the embryonic cuticle

It has previously been shown that cutinosomes carrying cuticular components start being deposited as patches at the globular stage and the cuticle becomes a continuous structure at heart stage (Stępiński et al., 2017; Creff et al., 2019; Coen et al., 2019; Doll and Ingram, 2022). Although the timing of cutinosome appearance and the early presence of the envelope suggests that the latter is not a cuticle-like structure, we tested this by analyzing mutants impaired in cuticle formation.

**Fig. 3.**
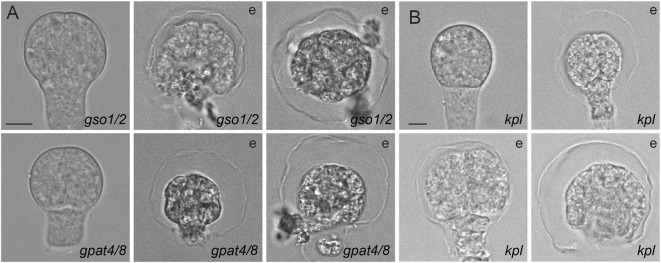
**The envelope is not a cuticle and is an embryo-derived structure.** (A) Embryos from cuticle-defective mutants, *gso1/2* and *gpat4/8*, showing the envelope (representative of *n*≥40 embryos per genotype). (B) *kpl* embryos developed in absence of the endosperm, showing the envelope (representative of *n*=40 embryos). e, after enzymatic treatment. Scale bars: 10 μm.

The GSO1 and GSO2 receptor-like kinases have been shown to be required for embryonic cuticle deposition. *gso1/2* double mutants display a patchy cuticle, and the embryos are physically attached to surrounding tissues (Tsuwamoto et al., 2008). We applied the same enzymatic treatment that liberated and allowed visualizing of the envelope to *gso1/2* embryos. We did not find a difference in the appearance of the envelope surrounding the proembryo and suspensor following enzyme treatment (Fig. 3A, upper panels). Additionally, we tested a double mutant in two *Arabidopsis* acyltransferases, GPAT4 and GPAT8. *gpat4/8* double mutants are deficient in cutin biosynthesis and more susceptible to desiccation and pathogen infection (Li et al., 2007). Embryos from these mutant lines displayed the envelope after enzymatic treatment (Fig. 3A, bottom panels). These results suggest that the embryo envelope is not an embryonic cuticle. We then tested the dynamics of enzymatic release of the envelope; we could not observe differences from the dynamics seen for the wild-type envelope (Fig. S3A).

### The envelope is likely to be derived from the embryo

It has been shown that the material to build the embryonic cuticle is secreted by the embryo (Moussu et al., 2013), whereas the material to build the embryonic sheath is delivered by the endosperm (Moussu et al., 2017; Doll et al., 2020). We asked which tissue might generate the embryo envelope. To determine its origin, we tested the presence of the envelope in the *kpl* (*kokopelli*) mutants in which single fertilization events predominate (Ron et al., 2010). The phenotypes observed in *kpl* mutants included ovules that were unfertilized, ones that underwent double fertilization, and ones that contained both embryo and developing endosperm, as well as those with products of a single fertilization, with a developing embryo and unfertilized central cell or with only developing endosperm (Ron et al., 2010). These different classes can be distinguished by size. Based on seed size, we isolated only embryos developing in the absence of the endosperm and performed an enzymatic treatment. We observed detachment of the embryonic envelope in *kpl* mutants (Fig. 3B), suggesting that the presence of endosperm is not required for envelope formation. Although it is theoretically possible that the unfertilized central cell generates the envelope, we conclude that crosstalk between the endosperm and the embryo is not needed for the deposition of the envelope. We tested the dynamics of the envelope liberation in *kpl* mutants and we did not observe differences from the wild-type (Fig. S3A). These results indicate that the embryonic envelope is likely built from components secreted by the embryo. Importantly, this also defines the envelope as a unique structure, distinct from the endosperm-derived embryonic sheath (Moussu et al., 2017).

### The embryonic envelope is enriched in glycoproteins and lipids

The embryonic envelope is observed as a thin layer covering the embryo surface and it can be partially detached from it by enzymatic treatment. The integrity of the envelope upon treatment with cell wall-degrading enzymes suggests that it is not a cell wall, and mutant analysis excludes that it is a cuticle. To investigate the composition of the envelope, we tested a range of established fluorescent dyes and antibodies. Given that staining with such dyes and antibodies at the surface of intact embryos would not allow us to differentiate between the envelope and underlying structures, we stained embryos that had first been treated with cell wall-degrading enzymes to release the envelope.

**Fig. 4.**
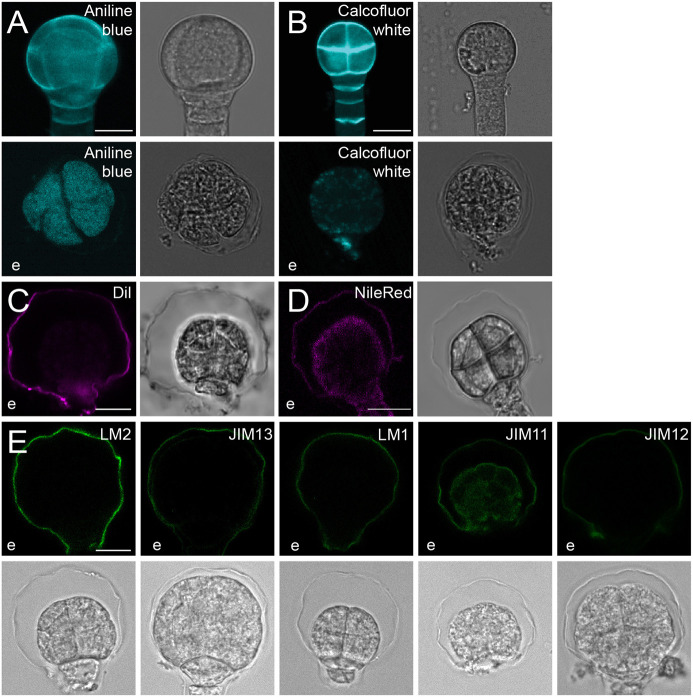
**Embryonic envelope composition.** (A,B) Calcofluor White (A) and Aniline Blue (B) staining of wild-type embryos (representative of *n*≥50 embryos per staining). (C,D) Wild-type embryos stained with DIL (C) and Nile Red (D) (representative of *n*≥8 embryos per staining). (E) Immunolabeling of wild-type embryos (representative of *n*≥30 embryos per staining) with LM2, JIM13, LM1, LM11 and LM12 antibodies. e, after enzymatic treatment. Scale bars: 10 μm.

First, we tested dyes that stain common components of the cell wall. Calcofluor White marks the primary cell wall polysaccharide cellulose (Wood, 1980; Mori and Bellani, 1996; Herburger and Holzinger, 2016) and, consistent with the results from enzymatic treatment, did not stain the embryonic envelope (Fig. 4A). Aniline Blue stains callose, a polysaccharide that is abundant in newly formed walls and plasmodesmata (Currier, 1957; Alexander, 1987; Zavaliev and Epel, 2015). No signal was observed in the envelope (Fig. 4A). We also checked for the presence of pectins by using LM20 (which binds to methyl esterified HG) and LM19 (which binds to unesterified HG) (Verhertbruggen et al., 2009). No signal was detected on the surface of the embryos, indicating that pectic polysaccharides are not present in this structure (Fig. S4). In contrast, the post-embryonic root surface was stained by both the LM19 and LM20 antibodies (Fig. S4).

We next used the lipid dyes DIL and Nile Red, which have been used before to stain different plant tissues, such as roots and leaves and in protoplasts (Diaz et al., 2008; Blachutzik et al., 2012; Li et al., 2015). Both dyes generated a clear fluorescent signal in the embryonic envelope (Fig. 4C,D), suggesting that lipids are a component of this structure.

In addition to polysaccharides and lipids, plant cells expose a rich landscape of extracellular proteins. Among these, two classes of *O*-glycoproteins are abundantly found in plant tissues: extensins (EXTs) and arabinogalactan proteins (AGPs) (Kieliszewski and Lamport, 1994; Kieliszewski et al., 2010; Ellis et al., 2010; Showalter et al., 2010). Importantly, the embryonic sheath is decorated by antibodies LM1 and JIM12, which label EXTs (Doll et al., 2020). We used a set of antibodies that label the glycosyl chains on hydroxylated proline residues in EXTs. LM1, JIM11 and JIM12 each recognize a fully glycosylated chain on EXTs (Willats and Knox, 2003; Knox, 2008; Moller et al., 2008). We used a whole-mount immunostaining method aimed at labeling the surface layer. As this method does not involve permeabilization steps, we expect little to no staining of the embryo itself, except if the envelope is ruptured, and antibodies can penetrate the embryo. All three antibodies clearly stained the envelope. Whereas LM1 and JIM12 appeared to be specific to the envelope, JIM11 also stained the embryo (Fig. 4E). Again, given that the method was designed to address surface labeling, we do not interpret this differential staining of the embryo.

We next used two antibodies that detect branched glycosyl groups on hydroxylated proline resides in AGPs. LM2 and JIM13 bind to β-linked glucuronic acid and (β)GlcA1→3(α)GalA1→2Rha epitopes, respectively (Willats and Knox, 2003; Knox, 2008; Moller et al., 2008). We found all antibodies to label the envelope (Fig. 4E). Thus, the envelope clearly stains positively for both EXTs and AGPs.

To determine whether we could correlate envelope composition to structure, we labeled *gso1/2* and *gpat4/8* mutant embryos with LM1 and LM2 antibodies to test for EXTs and AGPs epitopes, respectively (Fig. S5). Although the envelope is present in these mutants (Fig. 3A), they show defects in cuticle and sheath formation, and the latter in part labels for the same components detected on the envelope (Doll et al., 2020). We also analyzed embryos from *kpl* mutants, to test whether the absence of the endosperm affects the envelope composition. We found that LM1 and LM2 antibodies labeled the envelope in *gso1/*2, *gpat4/8* and *kpl* mutants*.* We quantified the fluorescence intensity on the envelope for each genotype and we could not detect significant differences between the mutants and the wild type (Fig. S5). Although it seems that the epitopes corresponding to AGPs are enriched, when compared to those from EXTs (Fig. S5) as AGP glycoproteins seem to be more prevalent than EXTs, this might suggest that AGPs are likely key components of the envelope.

### The envelope has properties of a polymeric structure

**Fig. 5.**
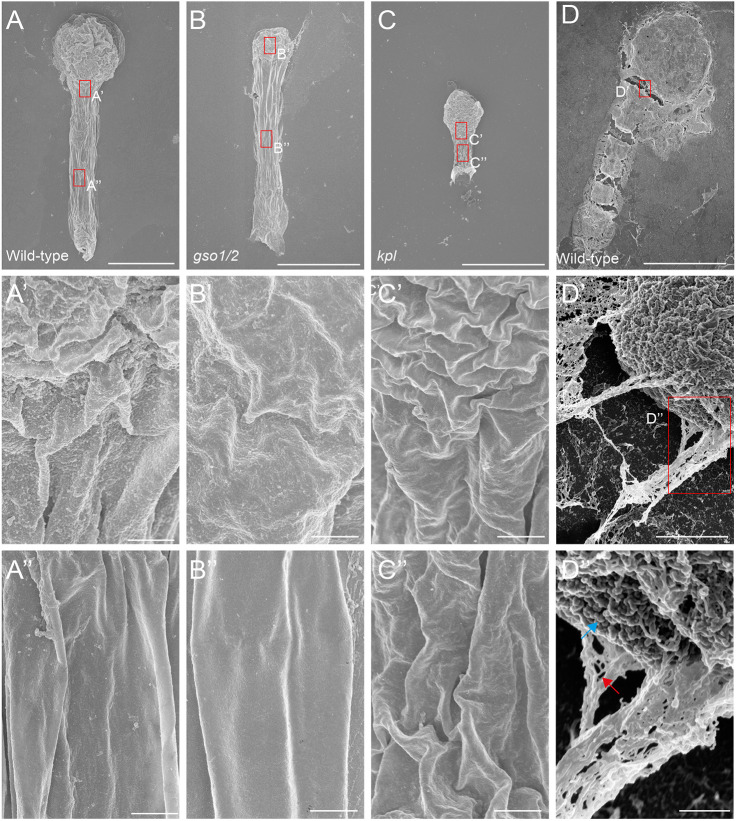
**SEM on *A. thaliana* embryos showing the surface structure of the envelope**. (A-C″) SEM on wild-type (A), *gso1/2* (B) and *kpl* (C) embryos without enzymatic treatment. The A, B and C panels are overview images of entire embryos; A′, B′ and C′ represent magnifications of surface of the proembryo, and A″, B″ and C″ are magnifications of suspensor surfaces. (D-D″) SEM on *Arabidopsis* wild-type embryo after enzymatic treatment. D′ and D″ represent magnifications of boxed area in D. Blue arrow points at globular structures and red arrow indicates filamentous features. All images are representative of *n*≥3 embryos for each genotype and treatment. Scale bars: 20 μm (A-C); 30 μm (D); 1 μm (A′,A″,B′,B″,C′,C″); 500 nm (D′,D″)

A remarkable feature of both AGPs and EXTs is that these proteins can be chemically crosslinked (Kjellbom et al., 1997; Brady and Fry, 1997; Brady et al., 1998), forming polymeric structures. Given that we identified these proteins as likely components of the envelope, we asked whether the envelope has properties that are consistent with crosslinked protein. To this end, we performed scanning electron microscopy (SEM) on isolated embryos that were either untreated or treated with cell wall-degrading enzymes. In control conditions (no enzymatic treatment) we observed the presence of a smooth continuous structure surrounding the entire embryo and suspensor (Fig. 5A; Fig. S6). Clearly, dehydration of the embryo during processing created a deflated embryo and wrinkled surface (Fig. 5A) that was decorated by a continuous structure following the entire wrinkled surface. We interpret this surface structure to be the envelope. As expected, the structure was comparable in *gso1/2* and *kpl* mutants (Fig. 5B,C). To probe the properties of the envelope, we analyzed enzyme-treated wild-type embryos. Although the overall structure and morphology of enzyme-treated embryos was affected by the treatment, we found that the surface structure persisted (Fig. 5D). In places where embryo cells had come apart during the treatment or processing, however, we observed thread-like filaments that connected the separated cells (Fig. 5D′,D″). Detailed imaging of these areas revealed strings of globular structures that were 35 nm in diameter. The characteristic diameter for individual globular proteins is 5 nm (Lobanov et al., 2008), indicating that the features we observed are unlikely to be linear protein filaments.

### Epidermal identity is required for embryonic envelope biogenesis

**Fig. 6.**
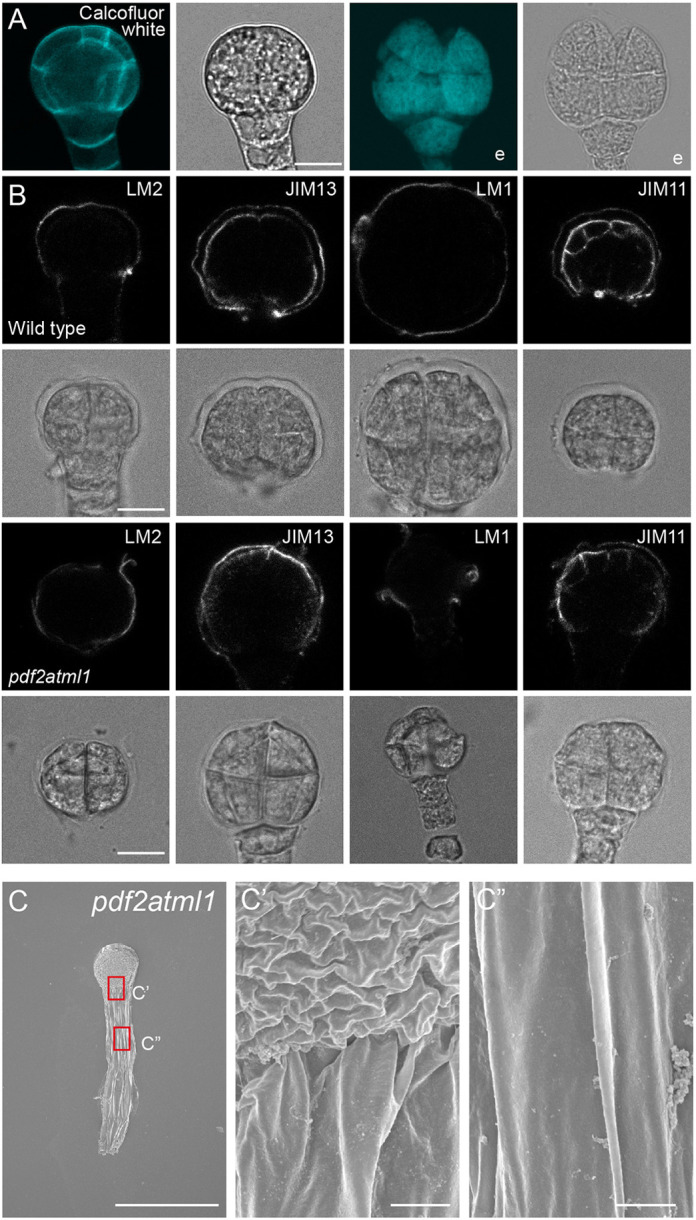
**Defects in epidermal cell fate specification affect the formation of the envelope.** (A) *pdf2 atml1* embryos stained with Calcofluor White either without or with enzymatic treatment (the latter denoted with an ‘e’). Images representative of *n*=30 embryos. (B) Immunolabeling of wild-type (top rows) and *pdf2 atml1* embryos (bottom rows) with LM2, JIM13, LM1 and JIM11 antibodies after enzymatic treatment (representative of *n*=50 embryos per genotype and antibody). (C) SEM of *pdf2 atml1* embryos in overview (C) and magnified views (C′,C″) (representative of *n*=4 embryos). Scale bars: 10 μm (A,B); 30 μm (C); 1 μm (C′,C″).

According to our results, the components needed to build the envelope are secreted by the embryo itself, but there are no known or suspected regulators of its production. However, given that the structure is formed on the surface, it is possible that its biogenesis is controlled by factors related to the epidermal identity of the surface-exposed cells. We therefore tested whether defects in epidermal cell fate specification could affect the formation of the envelope. We performed enzymatic treatment on embryos from *pdf2 atml1* mutants. Given that these double mutants are not viable or fertile as homozygous lines, we used segregating plants that were homozygous for one and heterozygous for the other mutation (*pdf2^−/−^ atml1^+/−^* and *pdf2^+/−^ atml1^−/−^*). We could observe the presence of the embryonic envelope in the mutants (Fig. 6A). Interestingly, however, the envelope appeared to be more damaged by the enzymatic treatment, showing discontinuities in its structure (Fig. 6A,B) that were not observed in wild-type embryos. This could be observed more in detail after the immunolabeling assays, in which a discontinuous fluorescent signal was observed (Fig. 6B). In these immunolabeling assays, we found that the main protein components, AGPs and EXTs, were still present in mutant envelopes.

To investigate whether the surface of the envelope showed any evident defects, we performed SEM on embryos from *pdf2 atml1* mutants but no clear differences were observed (Fig. 6C; Fig. S6). This could indicate that there are factors downstream the epidermal fate pathway affecting the composition of the envelope, which are more prone to break after the enzymatic treatment. Strikingly, we noticed that embryo cells appeared to dissociate from one another upon enzymatic treatment in the *pdf2 atml1* mutant (Fig. 6A,B), a phenomenon that was not observed in wild-type embryos. Thus, the altered properties of the envelope likely render it more permeable to cell wall-degrading enzymes.

## DISCUSSION

We have found a previously unreported thin membrane-like structure on the surface of early *A. thaliana* embryos. This embryonic envelope appears at early stages of development, and it can be detected until the late globular stage. To elucidate whether this structure could be an early embryonic cuticle, we analyzed *gpat4/8* and *gso1/2* embryos given that these mutants are defective in cutin synthesis and cuticle deposition, respectively (Tsuwamoto et al., 2008; Li et al., 2007). Embryos from both mutants displayed the thin continuous layer, showing that this structure is not the cuticle but rather an embryonic envelope. Another feature that distinguishes the envelope from the embryonic cuticle is that the former is found on the surface of the entire embryo whereas the latter is absent on the suspensor until the early and late heart stage (Szczuka and Szczuka, 2003). Moreover, *kpl* embryos developing in the absence of an endosperm display the envelope, showing that a crosstalk between the embryo and the endosperm is not required for the production of this structure. Our results show that the envelope is composed of crosslinked *O*-glycoproteins and lipids. These biomolecules could give the embryo envelope its properties, being flexible enough to accommodate changes during embryo growth and development. Moreover, analysis of *pdf2 atml1* embryos suggests that defects in the specification of epidermal cells leads to the production of an envelope that behaves differently after enzymatic treatment when compared to in the wild type. Although the components present remain largely the same, the embryonic envelope in these mutants seems to be more easily digested by the enzymes.

So far, we have not been able to assess the biological function of this structure, given the absence of a mutant that eliminates the envelope altogether. Raman imaging showed spectral peaks consistent with a proteinaceaous nature, but brief trypsin digestion of isolated embryos did not lead to the identification of major surface proteins (not shown). Therefore, the exact composition and key protein constituent of the envelope remains elusive, precluding genetic analysis. We can propose two hypotheses that will require deeper scrutiny in the future. First, the embryonic envelope could serve the same function as the sheath does in later developmental stages: forming an apoplastic diffusion barrier that both chemically and physically separates the developing embryo from the endosperm to allow both developmental programs to occur without unwanted crosstalk. Along the same lines, the envelope might serve as a template on which the cuticle and embryonic sheath are later assembled. Second, given the likely crosslinked proteinaceous nature of the envelope, the envelope could also serve a mechanical role. Plant developmental processes, in particular tissue patterning – which is crucial during embryogenesis (Palovaara et al., 2017; Harnvanichvech et al., 2021; Dresselhaus and Jürgens, 2021) – require a precise balance between outwardly directed osmotic forces, which are usually counterbalanced by tensile stresses in the stiff plant cell walls (Ali et al., 2023a,b). In early stages of embryogenesis, cell walls need to accommodate substantial growth and thus need to be plastic and malleable (Malinowski and Filipecki, 2002). The endosperm itself undergoes rapid developmental changes at the same time, which are associated with large decreases in turgor pressure (Fourquin et al., 2016), and thus also cannot continuously provide a mechanical counterforce to the action of the increasing embryonic turgor. The embryonic envelope could play a role in resolving this mechanical conflict. Later, at the heart stage and beyond, the deposition of the embryonic sheath by the endosperm starts, separating the embryo from the environment and preventing the developing tissues from sticking to the peripheral endosperm (Doll et al., 2020). At this stage, embryonic cell walls would be more capable of withstanding their own turgor, and the endosperm itself will have reached a more turgescent state (Fourquin et al., 2016) and so can offer an additional mechanical counterforce. Further analysis needs to be carried out to unravel the composition, nature and function, and phylogenetic distribution of the embryonic envelope.

## MATERIALS AND METHODS

### Plant material

*Arabidopsis thaliana* ecotype Columbia-0 (Col-0), *gpat4/8, gso1/2, kpl-2* and *pdf2-1 atml1-3* seeds were surface-sterilized and grown on ½ Murashige & Skoog (MS) medium (M0221, Duchefa) supplemented with 1% sucrose (w/v) and 0.8% plant agar (w/v) and stratified at 4°C for 2 days. Then, seeds were grown vertically on plates under long-day conditions (16 h light, 8 h dark) at 22°C for 2 weeks. Seedlings were transferred to soil and further grown at a constant temperature of 22°C under long-day conditions. Tomato (*Solanum lycopersicum*) and *Brassica napus* plants and cultures were kindly provided by Marian Bemer (Laboratory of Molecular Biology, Wageningen University and Research, The Netherlands) and Kim Boutilier (Bioscience, Wageningen University and Research, The Netherlands), respectively.

### Embryo isolation

Siliques were placed on double-sided tape and sliced open in 1× phosphate-buffered saline (1× PBS). Ovules from ∼20 siliques were collected in 2 ml Protein LoBind Tubes containing 20 μl of 1× PBS. Embryos were isolated with microcapillaries (VacuTipII, Eppendorf) and a micromanipulator (Eppendorf) under an inverted microscope (Carl Zeiss Microscopy) as described by Autran et al. (2011), and were washed once in 30 μl of 1× PBS.

### Enzymatic treatment

Embryos were incubated in 20 mM MES (pH 5.7) containing 0.4 M mannitol, 20 mM KCl, 2% (w/v) cellulase R10 (Duchefa, C8001), 0.5% (w/v) macerozyme R10 (Duchefa, M 8002), 0.05% (w/v) pectinase (Sigma-Aldrich, P2401), 0.05% (w/v) hemicellulase (Sigma-Aldrich, H2125) and 0.05% (w/v) Driselase (Sigma-Aldrich, D9515) for 20 min.

### Embryo envelope staining

After the enzyme treatment, embryos were incubated with 0.1% (w/v) Calcofluor White (18909, Sigma-Aldrich) or Aniline Blue (415049, Sigma-Aldrich) solutions for 5 min, and subsequently washed once with 1× PBS. For lipid staining, embryos were incubated with 0.1% (w/v) 1,1′-dioctadecyl-3,3,3′,3′-tetramethylindocarbocyanine perchlorate [DiIC18(3)] or Nile Red, and subsequently washed once with 1× PBS. For immunolabeling, embryos were incubated in 20 μl of 3% (w/v) milk protein in 1× PBS (MP/PBS) for 15 min to block non-specific binding sites. Embryos were then incubated with primary monoclonal antibodies (LM1 as18 4210, LM2 as18 4211, JIM11 as21 4692, JIM12 as21 4693 and JIM13 as22 4745; Agrisera) diluted 1:10 in MP/PBS for 15 min, and subsequently washed twice with 1× PBS. After washing steps, embryos were incubated with anti-mouse-IgG linked to fluorescein isothiocyanate (Sigma-Aldrich, F6258) diluted 1:100 in MP/PBS for 15 min. For quantification of the fluorescent signal, we used the segmented line tool in ImageJ to draw a line across the entire circumference of the envelope.

### Confocal microscopy

Images were acquired in 8-bit format using a Nikon C2 Confocal laser scanning microscope with 63× NA=1.20 oil-immersion objective with pinhole 1.0 Airy unit. Calcofluor White, Aniline Blue, DiIC18(3) and Nile Red were excited with 4 nm laser. Anti IgG-FITC was imaged in a SP8 Leica confocal microscope with 63× NA=1.20 water immersion objective with pinhole 1.0 Airy unit, using 488 laser and HyD SMD2 detector.

### TEM imaging

Details of the optimized procedure will be presented elsewhere. Briefly, immature seeds were high pressure-frozen and freeze-substituted then infiltrated with Spurr's resin and samples were transferred into BEEM capsules (Agar Scientific Ltd.) and polymerized at 70°C for 24 h. Polymerized blocks were trimmed into a pyramid shape and the face of the block was trimmed into a trapeze shape. Semi-thin (2 μm) and ultra-thin (70 nm) sections were cut with an ultra-microtome (Leica Ultracut, Leica Microsystems). The ultra-thin sections were post-contrasted in a LEICA EM STAIN (Leica Microsystems) with uranyl acetate (Polyscience Inc.) for 30 min followed by incubation in Reynolds' lead citrate (SERVA) for 90 s and subjected to TEM (JEOL JEM2100) at 2.00 kV.

### SEM imaging

Embryos were isolated as described above, placed on poly-lysine-coated glass slides, and allowed to settle at room temperature for 1 h without drying. Once the embryos were attached to the glass, they were gently rinsed with 1× PBS pH 7.4. A droplet of 2.5% glutaraldehyde was placed on the sample, and fixation was carried out for 1 h at room temperature. Samples were rinsed three times with 1× PBS. Post-fixation was carried out with 1% osmium tetroxide in phosphate buffer for 1 h at room temperature. Samples were rinsed three times with distilled water and dehydrated in an ethanol series (30%, 50%, 70%, 80%, 90%, 96% and 2×100% ethanol, each for 10 min). Samples were affixed to flat aluminum stubs using double-sided adhesive carbon tape. A 12-nm layer of tungsten was subsequently applied using a Leica EM SCD 500 sputter-coater. Finally, images were captured using an FEI Magellan 400 field-emission scanning electron microscope at an acceleration voltage of 2.0 kV.

## Supplementary Material

10.1242/develop.201943_sup1 Supplementary information
