# Supplementary material for: An elastic proteinaceous envelope encapsulates the early Arabidopsis embryo
Source: Development. 2023 Nov 9;150(22):dev201943. doi: 10.1242/dev.201943 (PMC10651100; doi:10.1242/dev.201943)
Supplement: Supplementary information [file develop-150-201943-s1.pdf]

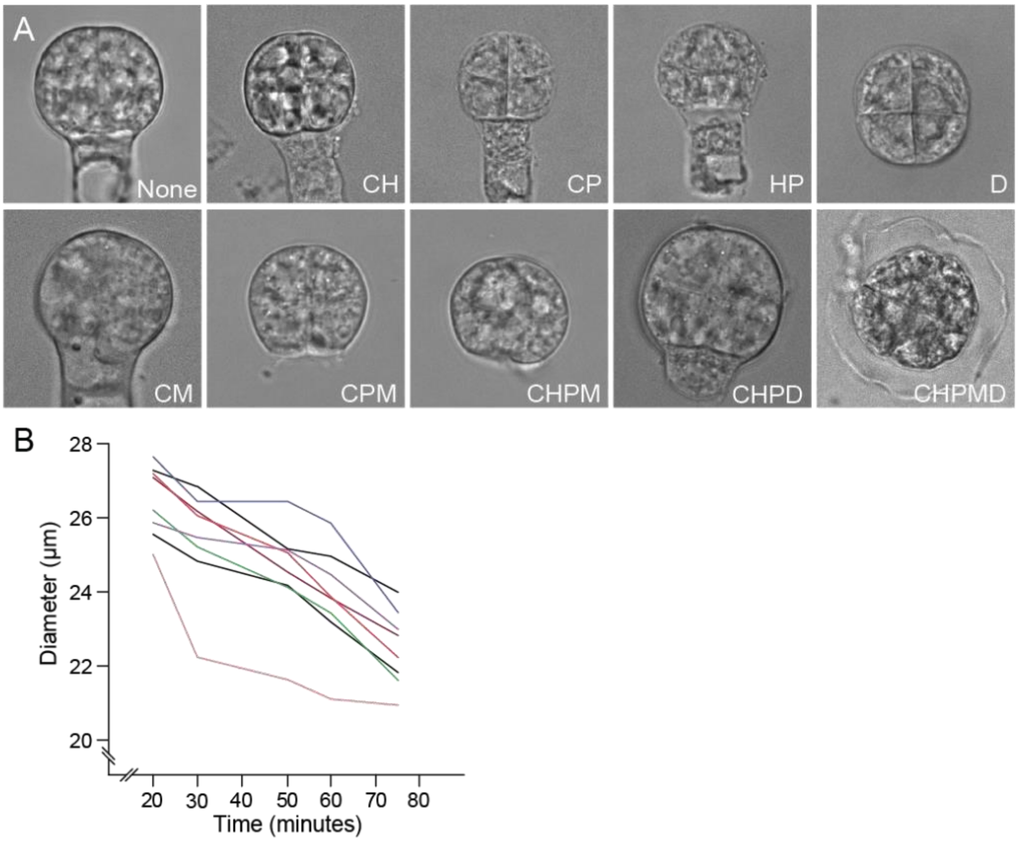

Fig. S1. Wild type embryos subjected to different enzyme combinations. (A) Morphology of embryos treated with different enzymes or mixtures thereof for 20 minutes (C, cellulose; H, hemicellulase; P, pectinase; D, driselase; M, macerozyme). (B) Quantification of embryo diameter during incubation with CHPMD enzyme mixture. Each trace shows the diameter of a single embryo.

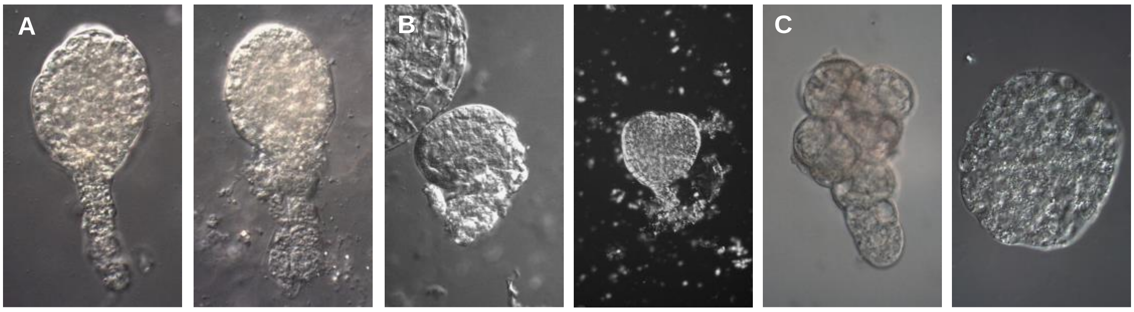

Fig. S2. Enzymatic treatment on embryos from *Solanum lycopersicum* and *Brassica napus*. (A-C) Enzymatic treatment on embryos from *S. lycopersicum, B. napus*, and *Brassica* microspore embryo cultures. For each image set the left panels and right panels correspond to images taken after 30 minutes and after 1h of enzymatic treatment, respectively.

Development • Supplementary information

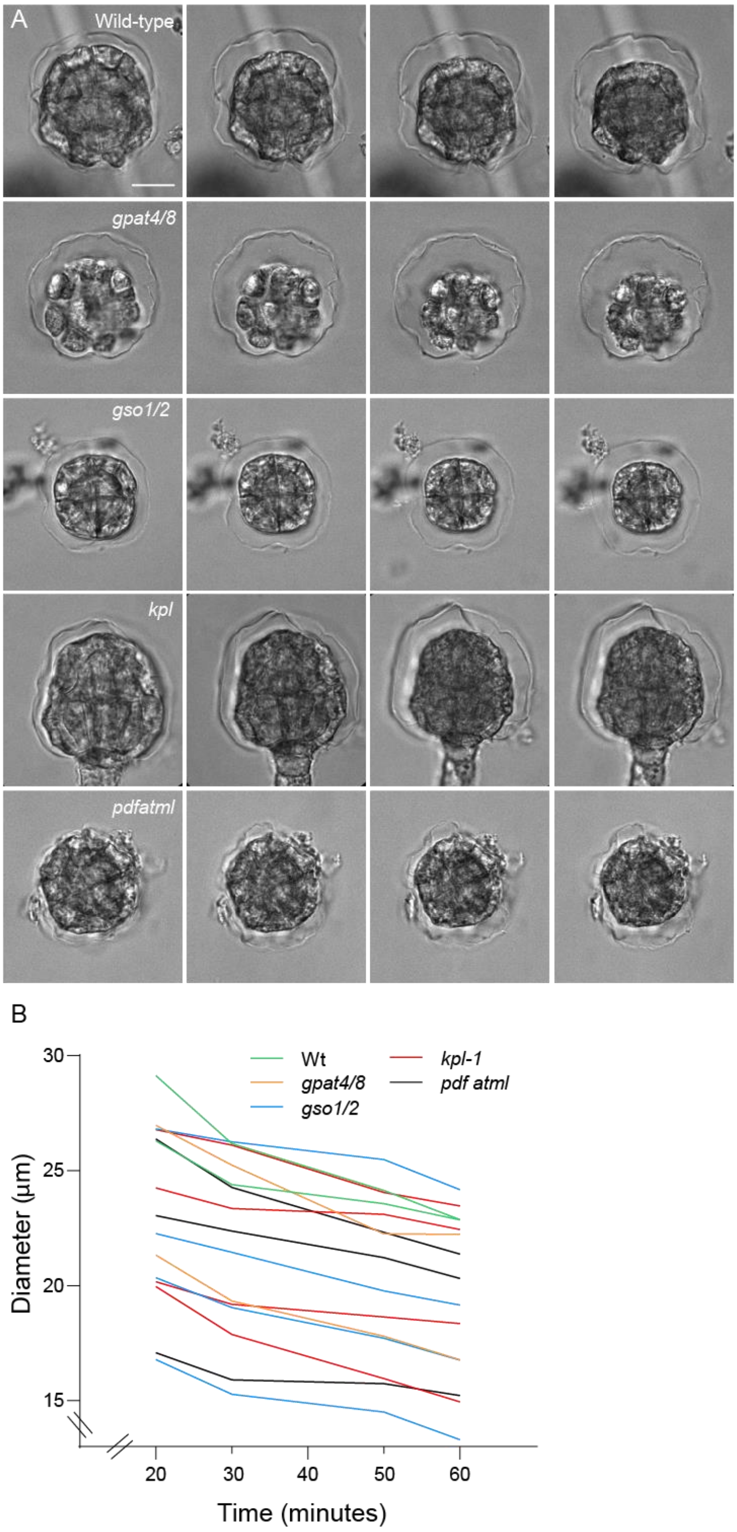

Fig. S3. Dynamics of envelope release.
A) Time course of the embryo envelope liberation. (B) Quantification of embryo diameter during incubation with CHPMD enzyme mixture. Each trace shows the diameter of a single embryo. Scale bar=10 µm.

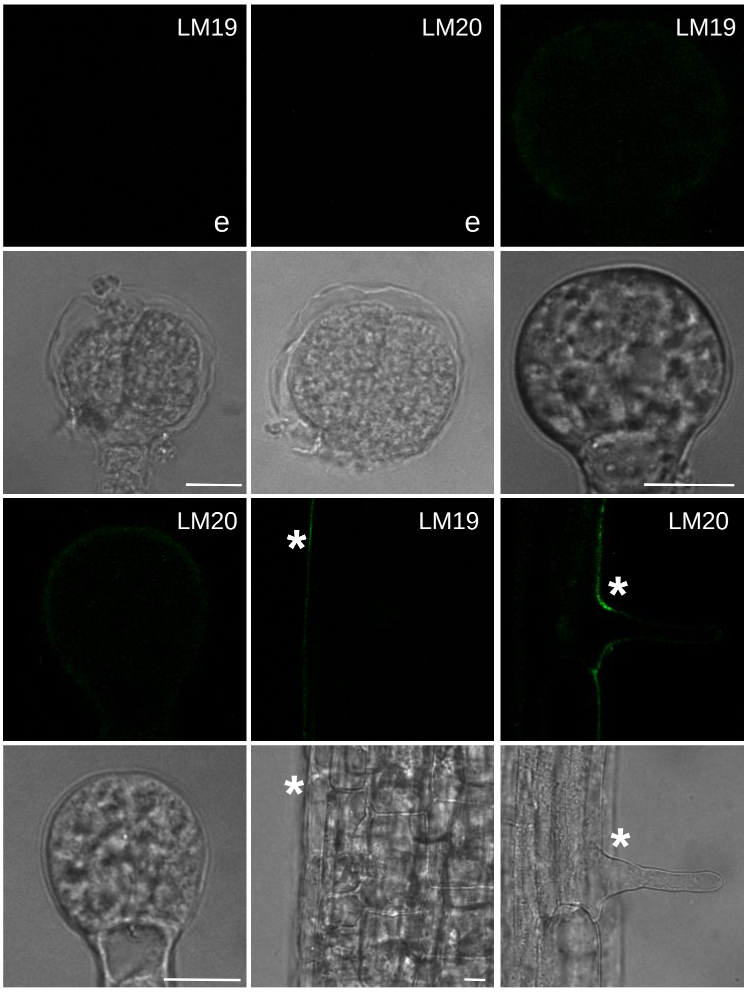

Fig. S4. Pectin immunolabeling.
Immunostaining of wild-type embryos (first 4 panels) and roots (last 2 panels) using LM19 and LM20 antibodies. “e” after enzymatic treatment. Asterisks mark positive signal at root surface. Scale bar=10 µm.

Development • Supplementary information

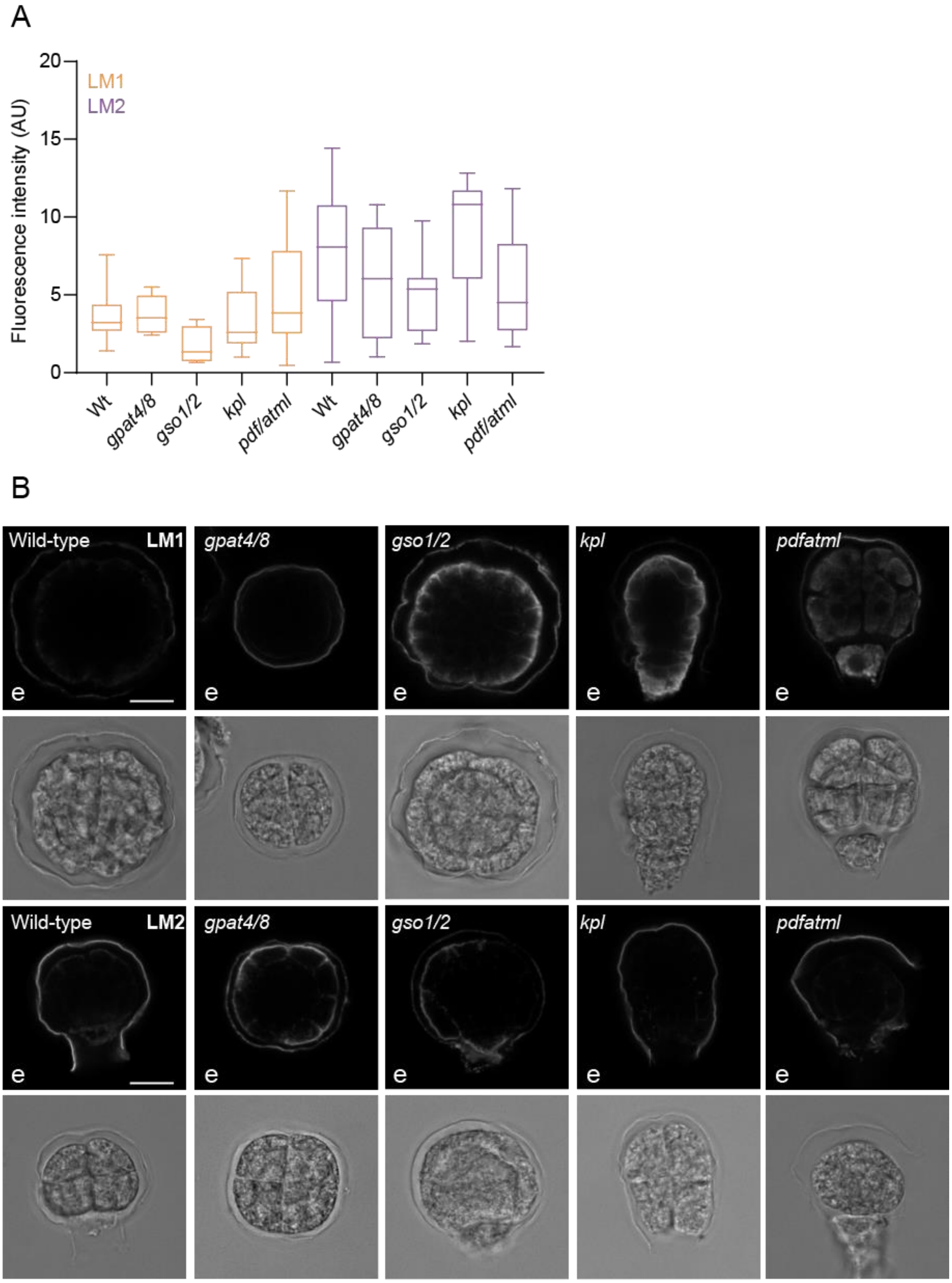

Fig. S5. Mutant embryo envelope immunolabeling.
(A) Quantification of immunostaining on gpat4/8, gso1/2 kpl and pdf2atml1 mutant embryos with LM1 and LM2 antibodies after enzymatic treatment (n≥20 embryos per antibody per genotype). (B) Representative images of each labeling in (A). Scale bar= 10 µm.

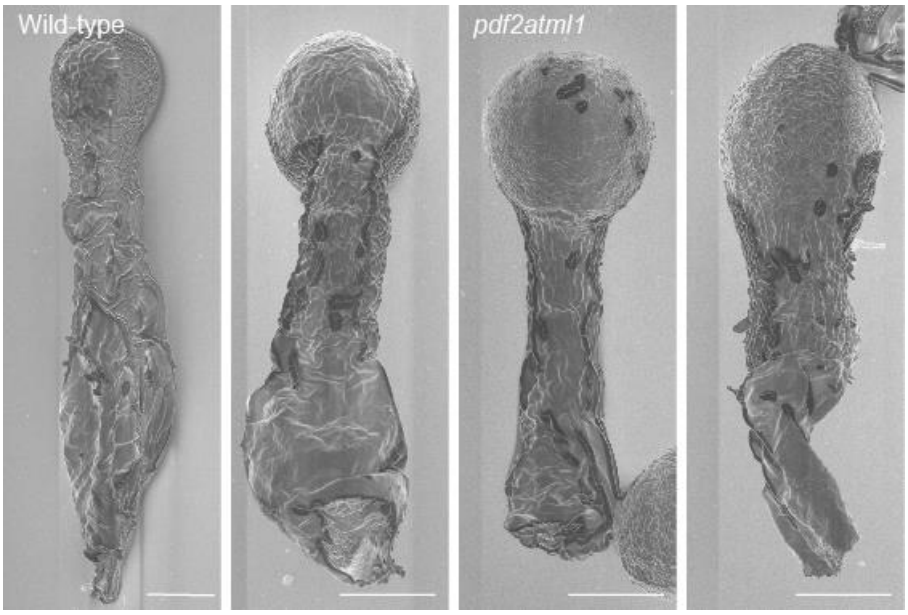

Fig. S6. SEM images of intact embryos.
The embryonic envelope can be observed on the surface of the proembryo and alongside the suspensor in wild-type and *pdf2atml1* embryos. Scale bars=10 µm.
