# Supplementary material for: An elastic proteinaceous envelope encapsulates the early Arabidopsis embryo
Source: Development. 2023 Nov 9;150(22):dev201943. doi: 10.1242/dev.201943 (PMC10651100; doi:10.1242/dev.201943)
Supplement: Supplementary file 1 [file develop-150-201943_review_history.pdf]

An elastic proteinaceous envelope encapsulates the early *Arabidopsis* embryo

Yosapol Harnvanichvech, Cecilia Borassi, Diaa Eldin S. Daghma, Hanne M. van der Kooij, Joris Sprakel and Dolf Weijers
DOI: 10.1242/dev.201943

Editor: Dominique Bergmann

Review timeline

Original submission:	3 May 2023
Editorial decision:	30 May 2023
First revision received:	9 October 2023
Accepted:	9 October 2023

Original submission

First decision letter

MS ID#: DEVELOP/2023/201943

MS TITLE: An elastic proteinaceous envelope encapsulates the early Arabidopsis embryo

AUTHORS: Yosapol Harnvanichvech, Cecilia Borassi, Diaa Daghma, Hanne van der Kooij, Joris Sprakel, and Dolf Weijers

I have now received all the referees' reports on the above manuscript, and have reached a decision. The referees' comments are appended below, or you can access them online: please go to BenchPress and click on the 'Manuscripts with Decisions' queue in the Author Area.

As you will see, the referees express considerable interest in your work, but have criticisms and recommend a revision of your manuscript that will require additional experiments before we can consider publication. In particular, further TEM studies, either to probe the components of the envelope (Rev 1) or in other species (Rev 3) are needed. If you are able to revise the manuscript along the lines suggested, I will be happy receive a revised version of the manuscript. Your revised paper will be re-reviewed by one or more of the original referees, and acceptance of your manuscript will depend on your addressing satisfactorily the reviewers' major concerns. Please also note that Development will normally permit only one round of major revision. If it would be helpful, you are welcome to contact us to discuss your revision in greater detail. Please send us a point-by-point response indicating your plans for addressing the refereeâ€™s comments, and we will look over this and provide further guidance.

Please attend to all of the reviewers' comments and ensure that you clearly highlight all changes made in the revised manuscript. Please avoid using 'Tracked changes' in Word files as these are lost in PDF conversion. I should be grateful if you would also provide a point-by-point response detailing how you have dealt with the points raised by the reviewers in the 'Response to Reviewers' box. If you do not agree with any of their criticisms or suggestions please explain clearly why this is so.

Reviewer 1

Advance summary and potential significance to field

© 2023. Published by The Company of Biologists under the terms of the Creative Commons Attribution License (https://creativecommons.org/licenses/by/4.0/).

The authors report their discovery of a previously undescribed proteinaceous, lipid containing, structure surrounding the developing embryo of Arabidopsis. They provide a globally convincing structural and genetic analysis of this structure, revealing that it is apparently distinct from two previously described embryo surface features: the cuticle and the embryo sheath.

Comments for the author

There are a few points that I would have liked the authors to address in more detail.

1) The authors say themselves that, given their data, they cannot categorically state that the structure that they observe in TEMs is the same at that that that they have analysed using dyes and antibodies after enzymatic release from the embryo surface. It's a bit of a shame that more effort was not made to nail this by performing immunogold labelling to try and localize the same epitopes seed in the released envelope, within TEM sections. I think it would be good to do this

2) Regarding the origin of the envelope. The authors use the fact that seeds issuing from crosses to the kpl mutant in which only the egg cell has been fertilized still produce the envelope, as an argument to suggest that it is entirely embryo derived, and that its production does not involve embryo-endosperm cross talk. To me this is a semantic trick (endosperm vs central cell) and the authors do, indeed, admit that the central cell could still be involved. To me this enigma (the origin of the structure) is thus unresolved. The fact that embryos with abnormal epidermal cell-fate specification show a defective envelope production would appear to support that premise that the embryo is important for envelope formation, but these mutants might have serious defects in embryo-endosperm cross talk (mediated by the epidermis). I think that again, using immunogold labelling to try and trace the origin of the components of the envelope might be a solution. If this is not possible, the authors should significantly tone down their conclusions.

3) Related to the above point. Would the authors be able to show the envelope in the basal most suspensor cell (in TEM of intact seeds)? Does the envelope really extend all the way around the embryo. Knowing this could help in addressing the above question?

4) The apparently "normal" envelope in mutants such as gso1/gso2, and kpl is very intriguing. I wonder whether the authors could go further with their assertion that these envelopes are, in fact, normal, by testing their enzymatic release dynamics? This would give valuable information regarding the whole surface structure of the embryo, and the anchoring of the envelope. It would also be really interesting to do this in the gpat4/8 mutant which, strangely, seems to have an abnormal composition?

5) The authors make some very interesting observations that never get properly discussed. For me one of the most important is the fact that gpat4/8 double mutants appear to lack labelling with EXTENSIN antibodies. This is very odd, especially since the expression of GPAT4 and 8 is likely dependent upon epidermal specification, but EXTENSINS seem to be present in the defective envelopes of atml1/pdf2 double mutants? Could the authors discuss this?

6) In Figure 5 D'', I cannot see "strings of globular structures". Please label.

More minor points

The figure legend in Figure 1 is clearly for a different version of the figure. In addition, it would be good to know at exactly what stage the thickness measurements were made?

The paper is really nicely written, with the exception of the introduction, which requires a little editing in order to read more smoothly.

For example:

Line 49- The embryo is not covered by the seed coat

Line 59- I don't think that the endosperm can be considered an "external" environment.

© 2023. Published by The Company of Biologists under the terms of the Creative Commons Attribution License (https://creativecommons.org/licenses/by/4.0/).

Reviewer 2

Advance summary and potential significance to field

In this paper, the authors described a newly identified structure named "embryonic envelope" that covers the surface of early embryos in Arabidopsis thaliala. Despite many years of Arabidopsis research, this structure has been overlooked in the previous observations. This paper is carefully written and includes sufficient information on the anatomy, composition, and origin of the embryonic envelope although the biological role of this structure still remains unknown.

Comments for the author

I have only minor comments.

1) I understand that it is technically difficult to manipulate the embryonic envelope during early embryogenesis. However, I feel laser ablation experiments would be useful to test whether the embryonic envelope can provide a mechanical counterforce to the developing embryos.

2) In figure legends, does "(n = 40 embryos)" mean 40 of 40 embryos showed the indicated phenotypes?

3) The embryonic envelope is still formed in the pdf2 atml1 embryo (even though it is prone to break). I feel that "its biogenesis depends on the determination of epidermal cell fate" may be misleading.

4) In the Figure 1 legend, (A) should be (A-C) and (B) should be (D). Scale bar information is missing.

Reviewer 3

Advance summary and potential significance to field

This manuscript presents a nice description of a new embryonic structure in Arabidopsis that the authors dub the embryonic envelop. They convincingly demonstrate that this structure precedes and is distinct from both the embryonic cuticle, as well as the embryo sheath, described by Ingram and colleagues.

I think the authors have delivered a rather complete histological description of the structure, using an extensive array of antibodies against a variety of cell wall epitopes. This is complemented by diverse treatments with cell wall digestive enzymes, as well as use of pertinent embryonic mutants.

I was a bit surprised that the authors do not present any attempt to check for the presence of similar structures in a few, select other plant species, such that they can make statements about the generality of their findings. I think this is not absolutely necessary, but would greatly increase the impact of their work.

I find the resistance of the embryonic envelope to a wide variety of enzymatic treatments rather striking, but would have hoped to see a treatment that would clearly digest the envelope. What about protease treatments? If the envelope is made of extensins and AGPs, at least in part, shouldn't that affect its structure?

I was sometimes not fully agreeing with the authors description of their results. First, some of the antibodies and stains give rather strong stains, others a really weak. LM2 is clearly giving a strong staining. In such a case, it would be much better not to use green or red LUTs, but intensity coded ones, that allows to better appreciate weaker signals. I had to increase my screen to maximum brightness to see some of the signals.

© 2023. Published by The Company of Biologists under the terms of the Creative Commons Attribution License (https://creativecommons.org/licenses/by/4.0/).

More importantly, I don't quite follow the authors conclusions about how the gso and gpat doubles are affecting the embryonic envelop. For example, the authors conclude that in gso1/2 all antibodies still stain - yes, but LM2 appears to be superweak, no? Is that a reproducible difference, or just a variability in the stainings? If the stainings are really variable, how do the authors intend todeal with this (comparison of wt in Fig. 4 and 6 indeed suggests quite a bit of variability, with JIM2 being much stronger than LM13 in Fig. 4, but as strong or weaker in Fig. 6). Could they provide some kind of straightforward quantification that give an impression of the variability? Or just show more pictures for each genotype in the supplements?

I am also really confused about the apparent absence of extensin stains in gpat doubles, not only because I would have thought that extensins should be really important for the structure of the envelop, but also because I thought that the embryonic cuticle is just starting to form at this stage. How do the authors think that gpat mutations affect embryonic envelop formation so early and strongly? Do they think GPATs directly participate in embryo envelop formation? Independently of their effect on cuticle? Did they look how early GPATs are expressed during embryogenesis?

Although the authors have not identified any mutants abrogating envelop formation and thus cannnot conclude about its importance, I think it is safe to assume that such a structure will be of functional significance and that the description of it is an important contribution to plant biology.

Comments for the author

I was a bit surprised that the authors do not present any attempt to check for the presence of similar structures in a few, select other plant species, such that they can make statements about the generality of their findings. I think this is not absolutely necessary, but would greatly increase the impact of their work.

I find the resistance of the embryonic envelope to a wide variety of enzymatic treatments rather striking, but would have hoped to see a treatment that would clearly digest the envelope. What about protease treatments? If the envelope is made of extensins and AGPs, at least in part, shouldn't that affect its structure?

I was sometimes not fully agreeing with the authors description of their results. First, some of the antibodies and stains give rather strong stains, others a really weak. LM2 is clearly giving a strong staining. In such a case, it would be much better not to use green or red LUTs, but intensity coded ones, that allows to better appreciate weaker signals. I had to increase my screen to maximum brightness to see some of the signals.

More importantly, I don't quite follow the authors conclusions about how the gso and gpat doubles are affecting the embryonic envelop. For example, the authors conclude that in gso1/2 all antibodies still stain - yes, but LM2 appears to be superweak, no? Is that a reproducible difference, or just a variability in the stainings? If the stainings are really variable, how do the authors intend todeal with this (comparison of wt in Fig. 4 and 6 indeed suggests quite a bit of variability, with JIM2 being much stronger than LM13 in Fig. 4, but as strong or weaker in Fig. 6). Could they provide some kind of straightforward quantification that give an impression of the variability? Or just show more pictures for each genotype in the supplements?

I am also really confused about the apparent absence of extensin stains in gpat doubles, not only because I would have thought that extensins should be really important for the structure of the envelop, but also because I thought that the embryonic cuticle is just starting to form at this stage. How do the authors think that gpat mutations affect embryonic envelop formation so early and strongly? Do they think GPATs directly participate in embryo envelop formation? Independently of their effect on cuticle? Did they look how early GPATs are expressed during embryogenesis?

Although the authors have not identified any mutants abrogating envelop formation and thus cannnot conclude about its importance, I think it is safe to assume that such a structure will be of functional significance and that the description of it is an important contribution to plant biology.

© 2023. Published by The Company of Biologists under the terms of the Creative Commons Attribution License (https://creativecommons.org/licenses/by/4.0/).

First revision

Author response to reviewers' comments

Response to reviewers
We are grateful for the supportive comments and feedback from all three reviewers. We have prepared a revision in which we have addressed all points raised to the best of our abilities. In our revised manuscript, we include a new set of experiments and text changes to clarify matters, or tone down conclusions, where relevant.
We believe that these revisions have helped to develop a more balanced manuscript. We respond to each point raised individually, below:

Reviewer 1:

1. *The authors say themselves that, given their data, they cannot categorically state that the structure that they observe in TEMs is the same at that they have analysed using dyes and antibodies after enzymatic release from the embryo surface. It's a bit of a shame that more effort was not made to nail this by performing immunogold labelling to try and localize the same epitopes seen in the released envelope, within TEM sections. I think it would be good to do this.*

We appreciate the reviewer's comments and suggestions, here we clarify the reasons why we could not such approach:
- For the presented samples in this manuscript, it is not possible to perform immunogold labeling because the samples were contrasted with OsO_4 (a strong oxidizing reagent) that blocks antigens in proteins. At temperatures higher than 0°C, OsO_4 acts proteolytically and the antigenicity is often destroyed (Roth, et al., 1981. J. Histochem. Cytochem. 29, 663–671. doi: 10.1177/29.5.6166664).
- Using low-temperature embedding media such as LR-white or HM20 was not possible in our hands as they are UV polymerized. Because embryos were fixed inside the seed coat and endosperm, UV light cannot penetrate sufficiently to cause polymerization and hardening of the resin so that samples can be sectioned.
- Immunogold labeling is possible in later Arabidopsis stages (heart stage and onward) when embryos can be isolated from the seeds. In our case, this would not be useful nor informative since at that stage the sheath is present (on top of the cuticle).

2. *Regarding the origin of the envelope. The authors use the fact that seeds issuing from crosses to the kpl mutant in which only the egg cell has been fertilized still produce the envelope, as an argument to suggest that it is entirely embryo derived, and that its production does not involve embryo-endosperm cross talk. To me this is a semantic trick (endosperm vs central cell) and the authors do, indeed, admit that the central cell could still be involved. To me this enigma (the origin of the structure) is thus unresolved. The fact that embryos with abnormal epidermal cell-fate specification show a defective envelope production would appear to support that premise that the embryo is important for envelope formation, but these mutants might have serious defects in embryo-endosperm cross talk (mediated by the epidermis). I think that again, using immunogold labelling to try and trace the origin of the components of the envelope might be a solution. If this is not possible, the authors should significantly tone down their conclusions.*

As we pointed out above, immunogold labeling was not an option for our samples. We have however toned down our conclusions regarding the origin of the envelope.

3. *Related to the above point. Would the authors be able to show the envelope in the basal most suspensor cell (in TEM of intact seeds)? Does the envelope really extend all the way around the embryo. Knowing this could help in addressing the above question?*

© 2023. Published by The Company of Biologists under the terms of the Creative Commons Attribution License (https://creativecommons.org/licenses/by/4.0/).

We appreciate this reviewer's comment and have performed SEM imaging in wild-type and *pdf atml* isolated embryos (no enzymatic treatment performed). This analysis showed that the envelope extends around the proembryo and suspensor. This information can be found in Supplementary figure 6.

4. *The apparently "normal" envelope in mutants such as gso1/gso2, and kpl is very intriguing. I wonder whether the authors could go further with their assertion that these envelopes are, in fact, normal, by testing their enzymatic release dynamics? This would give valuable information regarding the whole surface structure of the embryo, and the anchoring of the envelope. It would also be really interesting to do this in the gpat4/8 mutant which, strangely, seems to have an abnormal composition?*

We appreciate this reviewer's suggestion. We analyzed the envelope release dynamics for all the genotypes tested in this manuscript (wild-type, *gso1/2*, *gpat4/8*, *kpl* and *pfd atml*). We observed no significant differences in the extent to which the envelope detaches from the embryo in *gso1/2*, *gpat4/8*, *kpl* and *pdf atml* when compared to the wild-type. Although we could observe that for *pdf atml*, the morphology of the envelope is less sharp than in the other mutants tested. We also observed that, as in the wild-type, embryos shrink as a consequence of the treatment, and the structure becomes more distinctly visible with incubation time, yet does not dissociate from the embryo. **Supplementary figure 3.**

5. *The authors make some very interesting observations that never get properly discussed. For me one of the most important is the fact that gpat4/8 double mutants appear to lack labelling with EXTENSIN antibodies. This is very odd, especially since the expression of GPAT4 and 8 is likely dependent upon epidermal specification, but EXTENSINS seem to be present in the defective envelopes of atml1/pdf2 double mutants? Could the authors discuss this?*

We have found immunolabelling to be rather variable in intensity between samples and replicates. To be more specific on differences between genotypes, we repeated the experiments and quantified signals between genotypes. We limited this analysis to LM1 (EXTs labeling) and LM2 (AGP labeling). This analysis confirmed that labeling intensity is highly variable (see Supplementary Figure 5), and we therefore refrain from making strong statements about this.

6. *In Figure 5 D'', I cannot see "strings of globular structures". Please label.*

We have now annotated this panel with arrowheads to mark the structures indicated.

Reviewer 2:

7. *I understand that it is technically difficult to manipulate the embryonic envelope during early embryogenesis. However, I feel laser ablation experiments would be useful to test whether the embryonic envelope can provide a mechanical counterforce to the developing embryos.*

We considered this option, but realized that - given the tight association of the structure with the embryo - it would be impossible to perform the ablation without damaging the embryo itself.

8. *In figure legends, does "(n = 40 embryos)" mean 40 of 40 embryos showed the indicated phenotypes?*

"n" indicates the number of embryos analyzed. Given that we did not find observations deviating from the one shown, percentages are not given.

9. *The embryonic envelope is still formed in the pdf2 atml1 embryo (even though it is prone to break). I feel that "its biogenesis depends on the determination of epidermal cell fate" may be misleading.*

We have modified the conclusion to more accurately reflect the observations.

Reviewer 3:

10. *I was a bit surprised that the authors do not present any attempt to check for the presence of similar structures in a few, select other plant species, such that they can make statements about the generality of their findings. I think this is not absolutely*

© 2023. Published by The Company of Biologists under the terms of the Creative Commons Attribution License (https://creativecommons.org/licenses/by/4.0/).

necessary, but would greatly increase the impact of their work.

We had in fact done those experiments for the exact reason mentioned by the reviewer. However, revealing the structure requires its detachment from the embryo. We did not see a structure being liberated from tomato or Brassica zygotic embryos and Brassica microspore embryos (see Supplementary Figure 2). We find it hard to interpret this as negative evidence, since we can not be sure that the enzyme treatment is equally effective. We therefore leave this an open question in the manuscript.

11. *I find the resistance of the embryonic envelope to a wide variety of enzymatic treatments rather striking, but would have hoped to see a treatment that would clearly digest the envelope. What about protease treatments? If the envelope is made of extensins and AGPs, at least in part, shouldn't that affect its structure?*

We thank the reviewer for this comment. EXTs are crosslinked proteins, and therefore a protease treatment would not in fact dissolve the structure. Nevertheless, in one of our first attempts to digest the envelope, we treated the embryos with Trypsin, but did not succeed in detaching the envelope.

12. *I was sometimes not fully agreeing with the authors description of their results. First, some of the antibodies and stains give rather strong stains, others a really weak. LM2 is clearly giving a strong staining. In such a case, it would be much better not to use green or red LUTs, but intensity coded ones, that allows to better appreciate weaker signals. I had to increase my screen to maximum brightness to see some of the signals. More importantly, I don't quite follow the authors conclusions about how the gso and gpat doubles are affecting the embryonic envelop. For example, the authors conclude that in gso1/2 all antibodies still stain - yes, but LM2 appears to be superweak, no? Is that a reproducible difference, or just a variability in the stainings? If the stainings are really variable, how do the authors intend to deal with this (comparison of wt in Fig. 4 and 6 indeed suggests quite a bit of variability, with JIM2 being much stronger than LM13 in Fig. 4, but as strong or weaker in Fig. 6). Could they provide some kind of straightforward quantification that give an impression of the variability? Or just show more pictures for each genotype in the supplements?*

We have found immunolabelling to be rather variable in intensity between samples and replicates. To be more specific on differences between genotypes, we repeated the experiments and quantified signals between genotypes. We limited this analysis to LM1 (EXTs labeling) and LM2 (AGP labeling). This analysis confirmed that labeling intensity is highly variable (see Supplementary Figure 5), and we therefore refrain from making strong statements about this.

13. *I am also really confused about the apparent absence of extensin stains in gpat doubles, not only because I would have thought that extensins should be really important for the structure of the envelop, but also because I thought that the embryonic cuticle is just starting to form at this stage. How do the authors think that gpat mutations affect embryonic envelop formation so early and strongly? Do they think GPATs directly participate in embryo envelop formation? Independently of their effect on cuticle?*

Given the variability in staining we observed, we have toned down our conclusions regarding the *gpat* mutants.

14. *Did they look how early GPATs are expressed during embryogenesis?*

We have used our embryo transcriptome resource (https://albertodb.org/) to infer patterns of GPAT expression. See figure below. There is strong evidence for GPAT4 expression from early stages onward, while this is not clear for GPAT8. We have not included this information in the revision.

© 2023. Published by The Company of Biologists under the terms of the Creative Commons Attribution License (https://creativecommons.org/licenses/by/4.0/).

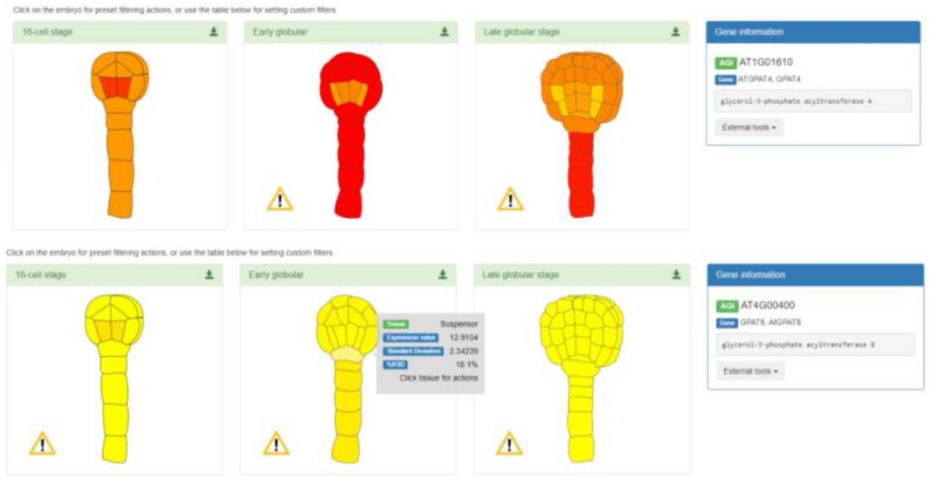

<u>Second decision letter</u>

MS ID#: DEVELOP/2023/201943

MS TITLE: An elastic proteinaceous envelope encapsulates the early Arabidopsis embryo

AUTHORS: Yosapol Harnvanichvech, Cecilia Borassi, Diaa Daghma, Hanne van der Kooij, Joris Sprakel, and Dolf Weijers
ARTICLE TYPE: Research Article

I am happy to tell you that your manuscript has been accepted for publication in Development, pending some minor text corrections andpending our standard ethics checks.

Specifically in the new text on page 6, there is a statement that the enzyme mixes do not digest linkages in these other species. This should either be changed to "we don't know whether the enzyme mix is capable of digesting the appropriate linkages in these other species", or you should provide the references or data for that statement. On page 9, new text has three highlighted supplementary figure call-outs, one of which has a placeholder X. Please put in the correct call-outs.

© 2023. Published by The Company of Biologists under the terms of the Creative Commons Attribution License (https://creativecommons.org/licenses/by/4.0/).
